# The Ideal Graduate Study Institution.—What Germany Has Done

**Published:** 1909-08-28

**Authors:** 


### Post-Graduate Medical Section.

### THE IDEAL GRADUATE STUDY INSTITUTION.—WHAT GERMANY HAS DONE.

### IV.—CONCLUSIONS AND THE MORAL.

In the preceding articles we have briefly sketched the rise and development of an organisation which, although not the oldest* post-graduate study association in Europe, must yet be considered as undoubtedly the best and most complete. We have thought it advisable to do so, because the Central Committee for the Promotion of Graduate Study in Prussia appears to us to have solved the problem of graduate study, and to have set about the work of organising such study in an ideal manner which is in every way worthy of imitation. Let us shortly recapitulate what that ideal consists of and what the means are by which it is carried out.

The main features of graduate study as carried on under the auspices of the Central Committee are that the courses are (1) both practical and theoretical, comprising all subjects of interest to the general practitioner and calculated to further his knowledge; (2) free; (3) given at times convenient for general practitioners engaged in private practice to attend; (4) and at places close to their sphere of practice; (5) with a membership limited to qualified men engaged in general practice. In addition, there is (6) a central main institute that serves as a bureau for information, as a domicile for special courses, and as a general centre for the whole movement.

1. The courses are practical and theoretical, and are given all the year round. Subjects are chosen of practical interest: the special work, of interest to the man with a hobby, is not made paramount, although practitioners can always obtain a course in some special and out-of-the-way subject. The theoretical courses, in the shape of lectures and demonstrations, are unlimited so far as membership is concerned. Practical work is done in small batches, which allows of individual assistance by the demonstrators and prevents crowding. Too much stress cannot be laid upon the fact that both special, practical, and theoretical courses are given by professors who are experts in their subjects, not by young docenten, who, however able to teach students, are hardly the men to lead a class of qualified men. One of the finest features of the organisation is the fact that men who have a world-wide reputation have come forward and volunteered to give up several hours a week for the benefit of their colleagues in general practice. Thus during the present year such courses were given by Professors von Renvers ("Novelties in Medical Diagnosis of Interest to the Practitioner"); Israels ("Points in Pathological Anatomy"); Körte ("Surgical Diagnosis"); Jansen ("Diseases of the Ear"); Silex ("Diseases of the Eyes"), and Eulenberg ("Mental Maladies in Private Practice"). The practical work, with demonstrations, is made interesting by means of the fine collection of demonstration specimens and the excellent apparatus with which the laboratories are fitted.

2. The courses are free; all that the practitioner has to pay is a "card fee" of two marks, which covers expenses of printing and secretarial work. Beyond this he has absolutely no expenses, except his personal outlay in tram fares. Apparatus, specimens, and the necessary outfit for each course are provided by the institution free of charge.

* The honour of having started the first post-graduate institution must belong, *mirabile dictu*, to the little-known and from a medical point of view interesting country Russia. At St. Petersburg exists the fine Helena Paulowna Post-Graduate Institution, founded in 1875, of which we hope to give a full description in a future issue.—Ed. The Hospital.

3. The hours for the courses are arranged to suit the convenience of members. At the first meeting of the class the professor or demonstrator suggests the time that would suit him, and invites suggestions from the class. These suggestions, often conflicting, are then amicably discussed, and a compromise arrived at which suits the majority. Here, as in other things, small sacrifices are inevitable, but the main sacrifice is usually on the part of the demonstrator.

4. The courses are held either at the Kaiserin Friedrich Haus or, where clinical demonstrations are required, at the hospitals in the vicinity. The central position of the institution suits this arrangement admirably, and hitherto the directors of the large city hospitals have worked harmoniously with the institution and given every facility to the general practitioner.

5 and 6. The advantages of the points included under these paragraphs need not be elaborated, as they are obvious to everyone.

Such, then, is the work of the Central Committee, and the English graduate who becomes acquainted with it can only envy his German colleague the opportunities and facilities which it affords the latter. The question remains whether he cannot try to imitate the work and to bring about in England a similar organisation, a similar central bureau, and similar facilities for graduate study. Discussing the matter with Professor Kutner, we pointed out the fact that the Polyclinic in London already to some extent does the work that is done by the Kaiserin Friedrich Haus. At the same time, we could not help calling to mind the points on which we had already laid stress in discussing the limitations of that institution. We there drew attention to the fact that the Polyclinic student lacks the opportunity for actual ward and clinical work, that the fees are comparatively high, and that the institution cannot at present be looked upon as a thoroughly representative one. The good points in the Polyclinic system were thoroughly grasped by our German colleagues, who in particular expressed their admiration of the system that permits the practitioner bringing his private patient for combined consultation and demonstration—a system which it is hoped to introduce in the German institution at an early date. "Surely," remarked Professor Kutner to us, "it would not be difficult, in so rich and generous a country as England, which supports its main hospitals by voluntary contributions, surely it would not be difficult to have an organisation and a central institution similar to the one here at Berlin and the institutions in Russia." It is a remark with which we heartily concur, and the success of the German movement should serve as an encouragement to those who look forward to the realisation of the hope that England in the near future may not be behind Germany and Russia in the development of graduate study in medicine.

At the International Medical Congress, now in session at Budapest, an important resolution will be brought forward by the Directors of the German and Russian Post-Graduate Associations. This resolution will voice the desire, felt by all graduate students, that the facilities for the prosecution of graduate study should be international, not local merely. Medicine belongs not to one country or one race; it is for all humanity, and the internationalisation of graduate study, the regular exchange of literature, information, and lists of courses, which is at present done spasmodically and without system, will do more to promote the good feeling and common interests between medical colleagues in various countries than the holding of congresses which are attended by specialists only. In going round the permanent exhibition in the Kaiserin Friedrich Haus we regretted the absence of British manufactures, English books, and any sign of British activity in the general advance of medicine. This omission can easily be rectified, and it will be in the interests of English medicine and English manufacturers to see that it is rectified as soon as possible. Equally important is it that British graduate study organisations should be represented at the Budapest Conference, to take their part in the discussion and to join the international committee for the promotion of graduate study in medicine and dentistry which it is proposed to form. We take it for granted that none of our readers will join issue with us on the statement that the promotion of graduate study in medicine and dentistry is a matter that is of primary importance in England as it has been found in other countries. The question of the relative value of the medical student's education is not one that need concern us here, for the fact remains that, no matter how well educated the student has been, he is unable to keep up his knowledge in the course of a busy life of private practice, or to remain in touch, unless he is connected as a teacher with one of the large medical schools, with the most recent advances in medicine. Equally true is it that a large number of general practitioners will gladly avail themselves of any opportunities offered them for increasing their knowledge, so long as these opportunities are given on lines similar to those which exist in Germany. That is to say, the three primary considerations that constitute the ideal—the courses should be absolutely free, given at times and places suitable for the man engaged in general practice, and essentially practitioners' and not students' courses—should be paramount. We need not labour these points, but it may be permitted here briefly to consider the manner in which such an ideal may be realised in England, and to take advantage of the lessons to be gained from a consideration of the German system.

The desirability of having a central institution which would serve as a general bureau and as the headquarters of any organisation, seems to us to be obvious. That such a central institution should be evolved out of any already existing organisation, associated with any particular hospital, college, or medical school is scarcely to be expected, nor, indeed, is it to be wished for. A movement in which the whole Empire should participate is not to be made to serve as a splendid advertisement for any particular institution or school; nor, again, is it to be localised in a place such as Oxford or Cambridge, whose associations with medicine are far less well known or generally recognised than those of our larger cities. The site for such an institution should be the Metropolis, but it should form branches, locally independent but in close touch with the headquarters, at various extra-metropolitan centres where sufficient material exists and sufficient interest is displayed to warrant the establishment of regular or periodical graduate courses. Such an institution, modelled on the lines of the Kaiserin Friedrich Haus in Berlin, and managed by as representative a board as that which looks after the latter institution, would serve many useful purposes, the enumeration of which need not be given here. Nor would its elaboration entail a colossal or extraordinary expense. The Berlin institution was built, equipped, and rendered practically self-supporting at a total cost of less than £60,000; the Helene Paulowna Institute was built and equipped at an even smaller sum, although it is annually receiving a State grant in its support. Making due allowance for the increased cost of labour and material in England, one may put the total cost of a similar institution in London at £100,000—a sum which is by no means excessive in view of the extended usefulness of such an institution. As in Berlin, the London institution with proper management can be made to be self-supporting. The question of State aid is one that we need not go into here, but we would merely point out that such an institution would deserve, for obvious reasons, the warm support of the Colonies and the equally cordial support of the various municipalities. At this time, when the medical inspection of school children is prominently before the public, and when, as we pointed out recently, the reform of the dental profession is imminent, the value of the services that such an institution can render to the nation is incalculable.

In order to bring it into being there is needed the interest of the medical profession more than an appeal to the generosity of the public. The main persons concerned directly in the promotion of graduate study are the practitioners themselves, and from them, and from the leaders of the profession must come the initiative in any movement that may lead to the establishment of such an organisation. The success which has been obtained in Germany would not have been won unless the medical profession there had energetically and actively interested themselves in the scheme, and by their endeavours stirred up the public to assist in the venture. On similar lines, any movement initiated here must be developed. We do not doubt that once such a movement is started, whole heartedly, sincerely, and unconnected with any particular association, hospital, or already existing body, it will achieve success and win for the general practitioner in England as fine an organisation and an institution as to-day exist in Germany. The proposal, made some years ago, to establish a post-graduate school in connection with a special academy for the study of comparative medicine is one which we energetically and emphatically must refuse to consider. We admit fully that a school of comparative medicine is an urgent desirability—though we do not admit that Cambridge or Oxford has a prior claim to it than London or Liverpool—but to make such a school an adjunct to an institution which must, if it wishes to adhere to the ideal of post-graduate study, rigidly exclude the unqualified student, is to stultify the usefulness of the latter. The separation of qualified from unqualified students in the German courses is one of the most praiseworthy features of the German system, and it is a feature which should be the dominant one in any system of graduate study.

Such a movement as we have attempted to outline is worthy the cordial support of Royalty. His Majesty the King and his Royal Highness the Prince of Wales have shown themselves in warm sympathy with the development of medical study, and have repeatedly given proof of their interest in matters connected with the profession. Their interest in the post-graduate study movement, seeing that it concerns no individual hospital or university but affects the mass of the profession, and indirectly the welfare of the whole community, will be an impartial and general interest which can only serve to encourage and stimulate those who are working to attain to the success which has crowned the German example.

---

Russian Military Tuberculosis Sanatorium in Finland.—According to *Helsingfors Hufvudstadsblad*, the Russian Ministry of War is erecting at Jerijoki, in the heart of the Finnish forest domains, a tuberculosis sanatorium for the men of the Russian regiments of the Guards, capable of accommodating five hundred patients; it is considered by high medical authorities in Russia that the balmy, dry, and resinous air of the Finnish pine-forests is highly beneficial in the arrest of this terrible disease.

